# The oncogenic fusion protein RUNX1-CBFA2T1 supports proliferation and inhibits senescence in t(8;21)-positive leukaemic cells

**DOI:** 10.1186/1471-2407-4-44

**Published:** 2004-08-06

**Authors:** Natalia Martinez, Bettina Drescher, Heidemarie Riehle, Claire Cullmann, Hans-Peter Vornlocher, Arnold Ganser, Gerhard Heil, Alfred Nordheim, Jürgen Krauter, Olaf Heidenreich

**Affiliations:** 1 Department of Molecular Biology, Institute for Cell Biology, Faculty of Biology, University of Tübingen, Auf der Morgenstelle 15, 72076 Tübingen, Germany; 2 Department of Haematology, Haemostaseology and Oncology, Hannover Medical School, 30625 Hannover, Germany; 3 Alnylam Europe AG, Fritz-Hornschuch-Str. 9, 95326 Kulmbach, Germany

## Abstract

**Background:**

The fusion protein RUNX1-CBFA2T1 associated with t(8;21)-positive acute myeloid leukaemia is a potent inhibitor of haematopoetic differentiation. The role of RUNX1-CBFA2T1 in leukaemic cell proliferation is less clear. We examined the consequences of siRNA-mediated RUNX1-CBFA2T1 depletion regarding proliferation and clonogenicity of t(8;21)-positive cell lines.

**Methods:**

The t(8;21)-positive cell line Kasumi-1 was electroporated with RUNX1-CBFA2T1 or control siRNAs followed by analysis of proliferation, colony formation, cell cycle distribution, apoptosis and senescence.

**Results:**

Electroporation of Kasumi-1 cells with RUNX1-CBFA2T1 siRNAs, but not with control siRNAs, resulted in RUNX1-CBFA2T1 suppression which lasted for at least 5 days. A single electroporation with RUNX1-CBFA2T1 siRNA severely diminished the clonogenicity of Kasumi-1 cells. Prolonged RUNX1-CBFA2T1 depletion inhibited proliferation in suspension culture and G1-S transition during the cell cycle, diminished the number of apoptotic cells, but induced cellular senescence. The addition of haematopoetic growth factors could not rescue RUNX1-CBFA2T1-depleted cells from senescence, and could only partially restore their clonogenicity.

**Conclusions:**

RUNX1-CBFA2T1 supports the proliferation and expansion of t(8;21)-positive leukaemic cells by preventing cellular senescence. These findings suggest a central role of RUNX1-CBFA2T1 in the maintenance of the leukaemia. Therefore, RUNX1-CBFA2T1 is a promising and leukaemia-specific target for molecularly defined therapeutic approaches.

## Background

The chromosomal translocation t(8;21) (q22;q22), which is associated with 10–15% of all cases of acute myeloid leukaemia, fuses the DNA binding domain of the transcription factor RUNX1 (also called AML1 or CBFα) to the almost complete open reading frame of CBFA2T1 (also named MTG8 or ETO) [1,2]. The resulting fusion protein RUNX1-CBFA2T1 (AML1/MTG8, AML1/ETO) interferes with haematopoetic gene expression by recruiting histone deacetylases via N-CoR and mSin3 to promoters, thereby inhibiting the transcription of the respective target gene [3-7]. Moreover, by directly binding to and sequestering transcription factors, such as SMAD3, C/EBPα or vitamin D receptor, RUNX1-CBFA2T1 interferes with signal transduction pathways controlling differentiation and proliferation [8-12]. Consequently, RUNX1-CBFA2T1 blocks myeloid differentiation and promotes self-renewal of haematopoetic progenitors [13-16].

The influence of RUNX1-CBFA2T1 on the control of proliferation and apoptosis is less clear. On the one hand, its ectopic expression in several cell types, including leukaemic cell lines such as U937, inhibits proliferation and enhances apoptosis [13]. On the other hand, RUNX1-CBFA2T1 may interfere with p53-dependent cell cycle arrest and apoptosis by suppressing the p53-stabilizing protein p14^ARF ^[17]. RUNX1-CBFA2T1 expression supports the expansion of haematopoetic progenitor cells, which has been mainly attributed to its anti-differentiation capabilities, but which may also depend on a proliferation supporting activity of RUNX1-CBFA2T1 [18-21]. RUNX1-CBFA2T1 alone is not sufficient to cause leukaemia [22,23]. Instead, secondary mutations have to be acquired in addition to RUNX1-CBFA2T1 to induce leukaemia [24-27].

Cellular senescence limits the proliferative capacity of cells and is characterized by an irreversible G1 arrest [28]. Senescent cells cannot be stimulated with mitogens to enter the S phase of the cell cycle. Nevertheless, senescent cells are still viable and metabolically active [29]. They can be distinguished from non-senescent cells by the expression of senescence-associated β-galactosidase activity, which can be detected at slightly acidic pH [30]. In the case of replicative senescence, cells enter G1 arrest after the telomeres have shortened below a critical length [29]. After exposure to stresses, cells may also undergo stress-induced senescence instead of apoptosis or transient cell cycle arrest [28]. The molecular mechanisms of senescence are still very incompletely understood. However, several regulators of cell cycle progression such as pRb, p53 or the cdk inhibitors p16^Ink4a ^or p27^Kip1 ^are involved in the establishment of senescence [31]. Furthermore, overexpression of oncogenic H-Ras in murine embryonic fibroblasts (MEFs) induce premature senescence in a PML-dependent fashion [32,33]. Similarly, overexpression of RUNX1 in MEFs induces senescence likely by upregulating p19^Arf ^[17]. However, control of senescence by an endogenously expressed oncogene such as RUNX1-CBFA2T1 in t(8;21)-positive leukaemic cells has not been shown yet.

The specific inhibition of gene expression by small interfering RNAs (siRNAs) provides a new approach to investigate the functions of oncogenes in the development of cancer, thereby complementing other approaches such as ectopic (over-) expression [34-36]. We and others have used siRNAs to specifically down-modulate leukaemic fusion genes such as BCR-ABL or RUNX1-CBFA2T1 [14,37-39]. We have shown that the siRNA-mediated depletion of RUNX1-CBFA2T1 led to a sensitization towards myeloid differentiation inducing agents such as TGFβ and vitamin D_3 _[14]. Here, we report the consequences of RUNX1-CBFA2T1 depletion on CD34 expression (a surface marker, indicator of differentiation), for the proliferation and clonogenicity of in t(8;21)-positive leukaemic cell lines. We demonstrate that RUNX1-CBFA2T1 supports the clonogenicity and proliferation of t(8;21) positive Kasumi-1 cells by interfering with the establishment of cellular senescence.

## Methods

### siRNAs

The siRNAs siAGF1 and siAM targeting the fusion site of the RUNX1-CBFA2T1 mRNA, the mismatch control siAGF6, and the unrelated controls targeting luciferase (siGL2) (for sequences see ref. 13) or MLL-AF4 (siMLL14; sense 5'-AAA CCA AAA GAA AAG CAG ACC-3', antisense 5'-GGU CUG CUU UUC UUU UGG UUU UU-3') were synthesized by either Alnylam Europe AG (Kulmbach, Germany) or MWG Biotech (Ebersberg, Germany) and hybridized as described [14].

### Cell culture and siRNA transfection

The t(8;21)-positive acute myeloid leukaemia (AML) cell lines Kasumi-1 (Deutsche Sammlung für Mikroorganismen und Zellkulturen (DSMZ) No. ACC 220) [40] and SKNO-1 [41], and the t(8;21)-negative leukaemic lines MV4-11 (DSMZ No. ACC 102), NB4 (DSMZ No. ACC 207), HL60 (DSMZ No. ACC 3), U937 (DSMZ No. ACC 5) and K562 (DSMZ No. ACC 10) were cultivated and electroporated together with siRNAs as described [14,42,43]. Briefly, cells were electroporated in 100–500 μl culture medium at a density of 5 × 10^6^/ml in 4 mm electroporation cuvettes. SiRNAs were added immediately before electroporation. If not otherwise indicated, the siRNA concentration during electroporation was 100 nM. Electroporations were performed using a rectangle pulse EPI 2500 electroporator (Fischer, Heidelberg, Germany; ). Parameters were 330 V and 10 ms (milliseconds) for Kasumi-1 cells, and 350 V and 10 ms (milliseconds) for all other cell lines. Fifteen minutes after electroporation, cells were diluted twenty fold in culture medium and incubated at 37°C, 5% CO_2 _and 92% humidity. Using this protocol, we routinely observe less than 10% of dead cells after electroporation with an siRNA transfection efficiency close to 100% [14,42,43].

### Immunoblotting

To obtain total cell lysates, cells were washed with phosphate-buffered saline, lysed in Urea lysis buffer (9 M urea, 4% (w/w) 3-[(3-Cholamidopropyl)-dimethylammonio]-propansulfonat (CHAPS), 1% (w/w) Dithiothreitol) and treated for 30 min on ice with 2.5 u/ml Benzonase (Merck, Darmstadt, Germany). Total (10–20 μg) and nuclear lysates (10 μg) were analyzed as described [14]. Band intensities were determined by scanning the exposed films followed by quantification using the Image Gauge 3.0 software (Fujifilm, Düsseldorf, Germany). The following antibodies were used for detection: AML1/RHD (2.5 mg/l; Oncogene Research PC285, Boston, USA); p27 C-19 (200 μg/l; Santa Cruz Biotechnology sc-9737; Heidelberg, Germany); Tubulin Ab-4 (1 mg/l, NeoMarkers MS-719-P0, Fremont, USA).

### Real-time RT-PCR

Total RNA was isolated using the RNeasy Kit (Qiagen, Hilden, Germany). Reverse transcription was performed with random hexamers using MMLV-RT, RNase H^- ^(Promega, Heidelberg, Germany) as suggested by the manufacturer. Real time PCRs were performed using Sybr-Green Mix (Applied Biosystems, Warrington, UK), 300 nM primers and 20% v/v diluted RT reaction mix using standard conditions on a 7000 Sequence detection system (Applied Biosystems, Foster City, CA, USA). The primer sequences for STAT1 (5'-CAT CAC ATT CAC ATG GGT GGA (forward primer) and 5'-GGT TCA ACC GCA TGG AAG TC (reverse primer)), for CD34 (5'-TCC AGA AAC GGC CAT TCA G (forward primer) and 5'-CCC ACC TAG CCG AGT CAC AA (reverse primer)), for G-CSF (5'-CCC ACC TTG GAC ACA CTG C (forward primer) and 5'-AGT TCT TCC ATC TGC TGC CAG (reverse primer)) and for GAPDH (5'-GAA GGT GAA GGT CGG AGT C (forward primer) and 5'-GAA GAT GGT GAT GGG ATT TC (reverse primer)) were designed with Primer-Express software (Applied Biosystems, Foster City, CA, USA).

### Proliferation, single cell expansion and colony formation assays

To determine proliferation rates and doubling times, cells were counted using trypan blue. For single cell expansion assays, 24 h after electroporation cells were diluted to a density of 10 cells/ml and plated in 100 μl aliquots into 96 well tissue culture plates. Colony formation assays were performed 24 h after electroporation with a density of 5,000 cells/ml in semisolid medium (RPMI1640 containing 20% fetal calf serum and 0.5625% methyl cellulose). Colonies were counted 14 days after plating. The cytokine concentrations were 20 ng/ml for G-CSF and GM-CSF in the cell cycle and senescence assays, and 10 ng/ml for all growth factors in the colony formation assays.

### Cell cycle analysis

Cell cycle analysis was performed as described [44]. Four days after electroporation, 10^6 ^cells were suspended in 200 μl citrate buffer (250 mM sucrose, 40 mM sodium citrate pH 7.6) followed by the addition of 800 μl staining solution (phosphate-buffered saline containing 20 mg/l propidium iodide, 0.5% (w/w) NonIdet P40, 500 μM EDTA) and 10 μl boiled RNase A (10 g/l). Cells were kept for 30 minutes on ice and analyzed by flow cytometry (FACSCalibur, Becton Dickinson, Heidelberg, Germany). Data analysis was performed with FCSPress 1.3 .

### Analysis of apoptosis and of CD34 expression

For quantification of apoptotic cells, cells were stained with FITC-labeled annexin V as suggested by the supplier (Bender, Vienna, Austria). CD34 expression was monitored by staining with anti-human CD34-FITC (clone 581, BD Pharmingen #555388, Heidelberg, Germany). Analysis was performed by flow cytometry (FACSCalibur, Becton Dickinson, Heidelberg, Germany) using FCSPress 1.3  for data analysis.

## Results

### Time course of RUNX1-CBFA2T1 protein depletion by siRNAs

**Figure 1:**
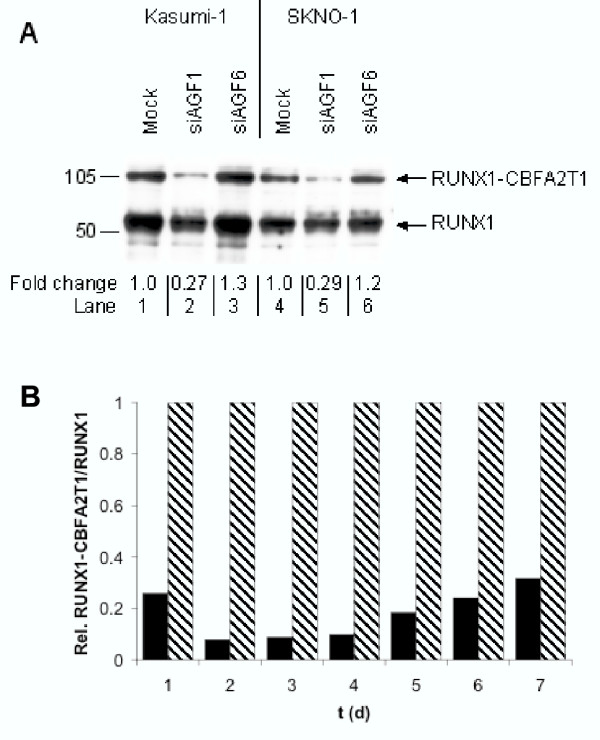
***Depletion of RUNX1-CBFA2T1 protein by siRNAs ***A. RUNX1-CBFA2T1 and RUNX1 protein levels in siRNA-treated Kasumi-1 and SKNO-1 cells. Nuclear lysates were prepared 4 days after electroporation with 100 nM siRNAs and analyzed by immunoblotting. The electroporated cells and siRNAs are indicated on top. Arrows on the right mark RUNX1-CBFA2T1 and RUNX1 proteins. Markers are shown on the left, and the relative ratios between RUNX1-CBFA2T1 and RUNX1 are indicated below the blot. B. Time course of siRNA-mediated RUNX1-CBFA2T1 depletion. Kasumi-1 nuclear lysates were isolated at the indicated days after electroporation with 100 nM siRNA and analyzed by immunoblotting. Values were normalized to the control siRNA siAGF6.

Electroporation of t(8;21)-positive cells with the RUNX1-CBFA2T1 siRNA siAGF1, but not with the mismatch control siRNA siAGF6, resulted in a three- to fourfold reduction of RUNX1-CBFA2T1 fusion protein in both Kasumi-1 cells (fig. 1A, lanes 1–3) and SKNO-1 cells (fig. 1A, lanes 4–6). One day after electroporation with siAGF1, RUNX1-CBFA2T1 protein levels were already reduced by 75% (fig. 1B). This finding suggests that RUNX1-CBFA2T1 protein has a half-life of less than 24 hours. The depletion of RUNX1-CBFA2T1 lasted for at least 7 days with a slow recovery of RUNX1-CBFA2T1 protein levels from day 5 on (fig. 1B), in agreement with the previously reported time-course of siRNA-mediated RUNX1-CBFA2T1 mRNA reduction [14].

Transfection with siRNAs may induce an interferon response leading to non-specific effects on cellular processes such as proliferation or differentiation [45]. Since STAT1 is an interferon-stimulated gene, and since several siRNAs have been shown to induce STAT1 expression [45], we examined a possible induction of STAT1 gene expression by RUNX1-CBFA2T1 siRNAs or by mismatch control siRNAs in Kasumi-1 cells. Neither the electroporation with RUNX1-CBFA2T1 siRNA nor with the control siAGF6 caused a substantial change in STAT1 mRNA levels (data not shown), suggesting that these siRNAs do not induce an interferon response in Kasumi-1 cells.

### RUNX1-CBFA2T1 siRNAs decrease the clonogenic growth of Kasumi-1 cells

**Figure 2:**
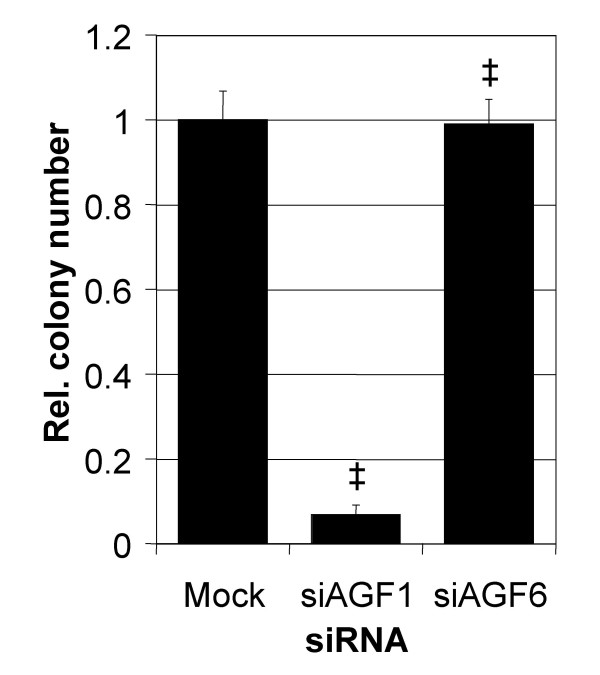
***RUNX1-CBFA2T1 depletion reduces the clonogenicity of Kasumi-1 cells ***The columns represent the means of four independent experiments. Error bars indicate standard deviations. ‡, p < 0.001 according to Student's t-test.

In comparison to mock-transfected cells, electroporation with RUNX1-CBFA2T1 siRNAs resulted in a ten- to twenty fold decreased clonogenicity in both single cell expansion (data not shown) and colony formation assays (fig. 2). Electroporation with the mismatch control siRNA siAGF6 affected the clonogenicity in neither of these assays. We did not observe major differences in colony morphology. However, the colonies derived from cells treated with RUNX1-CBFA2T1siRNAs tended to be smaller in size than those derived from control cells (data not shown).

We also examined the effects of RUNX1-CBFA2T1 siRNA on the clonogenicity of AML cell lines not expressing RUNX1-CBFA2T1. Colony formation of the t(8;21)-negative myeloid leukaemia cell lines MV4-11, NB4, HL60, U937 and K562 was not affected upon electroporation with RUNX1-CBFA2T1 siRNA (data not shown), which argues against a general, unspecific effect of the siRNAs used in this study on leukaemic clonogenicity. Therefore, the application of RUNX1-CBFA2T1 siRNAs is sufficient to inhibit the clonogenic growth of Kasumi-1 cells, indicating that RUNX1-CBFA2T1 is essential for the clonogenicity of these leukaemic cells.

### RUNX1-CBFA2T1 depletion reduces CD34 expression

**Figure 3:**
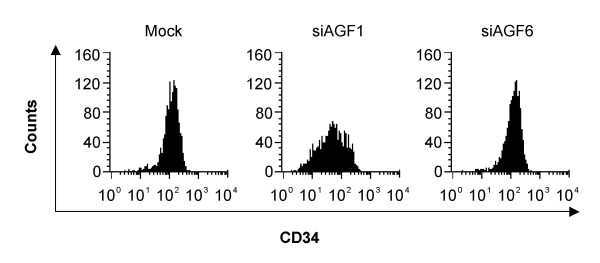
***RUNX1-CBFA2T1 depletion diminishes CD34 expression ***Seven days after electroporation with the indicated siRNAs, cells were analyzed for CD34 surface marker by flow cytometry as described in Material and Methods.

To examine the effects of RUNX1-CBFA2T1 on the progenitor status of t(8;21)-positive leukaemic cells, we analyzed the CD34 expression levels in siRNA-treated Kasumi-1 cells. When compared to mock-transfected cells, depletion of RUNX1-CBFA2T1 caused a twofold decrease in CD34 surface expression 7 days after electroporation (fig. 3). Control siRNA-treated cells contained only slightly lower amounts of CD34 surface marker.

### RUNX1-CBFA2T1 suppression inhibits proliferation in suspension cell culture

**Figure 4:**
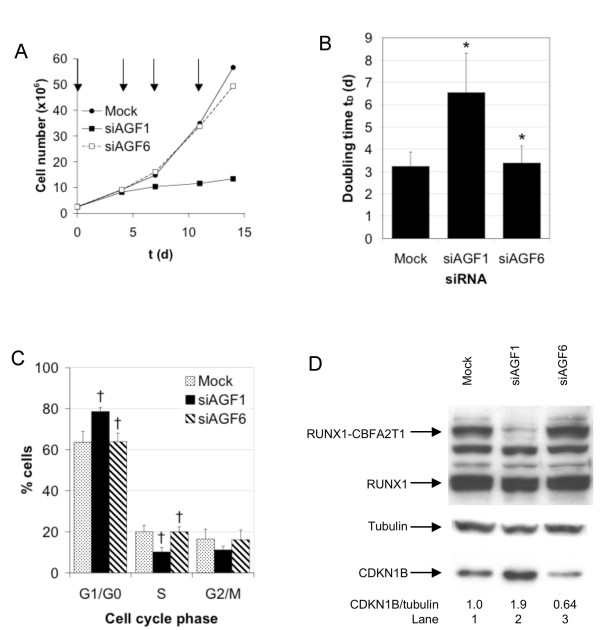
***RUNX1-CBFA2T1 depletion inhibits cell proliferation and G1-S transition in Kasumi-1 ***A. Proliferation curve of siRNA treated cells. Kasumi-1 cells were electroporated at days 0, 4, 8 and 12. Cell numbers were determined at the indicated days using trypan blue counting. Arrows indicate electroporations. B. Graphical representation of cell doubling times. The columns represent the means of three independent experiments. Error bars indicate standard deviations. *, p < 0.05 according to Student's t-test. C. Graphical representation of the cell cycle phase distribution. Kasumi-1 cells were electroporated at days 0 and 4, and were examined at day 8 using FACS analysis. The columns and error bars represent the mean values and standard deviations of three independent experiments. †, p < 0.01 according to Student's t-test. C. RUNX1-CBFA2T1 suppression is associated with increased CDKN1B (p27^Kip1^) levels. After electroporation at days 0 and 4, total cell lysates were prepared at day 8 and analyzed using immunoblotting. After CDKN1B detection, the membrane was stripped and reprobed with an anti-tubulin antibody. The siRNAs are indicated on top. Arrows on the left mark RUNX1-CBFA2T1, RUNX1, CDKN1B (p27^Kip1^) and tubulin proteins. The relative ratios between CDKN1B and tubulin are indicated below the blot.

In contrast to its effects on clonogenicity, a single electroporation with RUNX1-CBFA2T1 siRNA was not sufficient to substantially inhibit Kasumi-1 proliferation in suspension culture. However, repeating the electroporation every three to four days strongly inhibited cell proliferation (fig. 4A). During a course of three to four electroporations with or without mismatch control siRNA, the cell numbers steadily increased with a doubling time of 3 days (fig. 4B). RUNX1-CBFA2T1 siRNA treatment increased the doubling time twofold. Therefore, repetitive siRNA applications causing a long-lasting RUNX1-CBFA2T1 depletion inhibited cell proliferation of Kasumi-1 cells. These results suggest that RUNX1-CBFA2T1 is essential for the proliferation of t(8;21)-positive leukaemic cells in suspension culture.

### RUNX1-CBFA2T1 suppression interferes with cell cycle progression

Next, we examined possible consequences of RUNX1-CBFA2T1 depletion for the cell cycle distribution of Kasumi-1 cells. Three days after an electroporation with RUNX1-CBFA2T1 siRNA we observed 25% less cells being in S phase (data not shown). Two or more consecutive electroporations with RUNX1-CBFA2T1 siRNA caused a reduction of the amount of S phase cells by 50% and a 30% reduction in G2/M phase cells, with a corresponding increase of the G1/G0 fraction by 20% (fig. 4C). Treatment of Kasumi-1 cells with the mismatch control siRNA did not affect the cell cycle distribution.

The changes in the cell cycle distribution are associated with changed expression levels of CDKN1B (p27^Kip1^), a general inhibitor of Cyclin-CDK complexes and, thereby, of cell cycle progression. Two electroporations with RUNX1-CBFA2T1 siRNA caused a twofold increase in CDKN1B protein levels in RUNX1-CBFA2T1-depleted cells, but not in control cells (fig. 4D). Therefore, siRNA-mediated inhibition of RUNX1-CBFA2T1 expression may interfere with the transition of Kasumi-1 cells from the G1 to the S phase of the cell cycle.

### RUNX1-CBFA2T1 controls apoptosis

**Figure 5:**
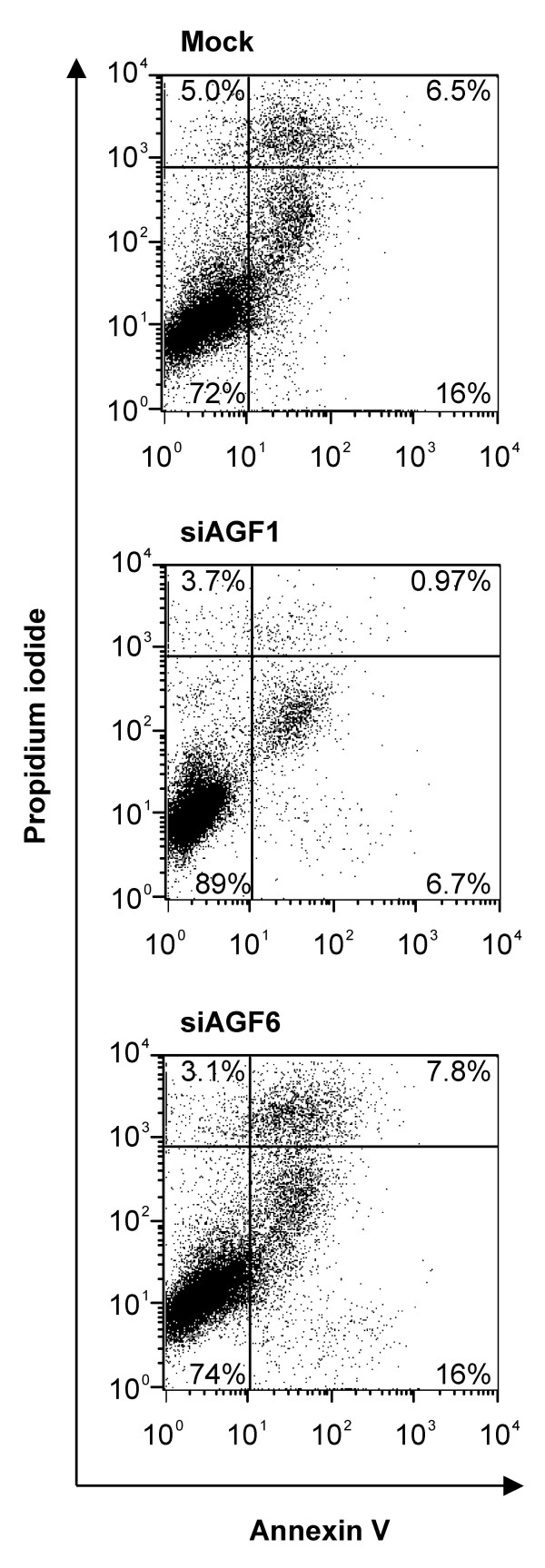
***Effects of RUNX1-CBFA2T1 and RUNX1 suppression on extent of apoptosis in Kasumi-1 cells ***Kasumi-1 cells were electroporated at days 0 and 4, and were examined at day 8 by annexin V and propidium iodide staining followed by FACS analysis. One of two experiments yielding very similar results is shown. The percentage of cells is given for each quadrant of the plots.

A possible reason for the observed inhibition of proliferation is the induction of apoptosis upon suppression of RUNX1-CBFA2T1. We addressed this possibility by examining the amount of apoptotic cells by annexin V staining and of hypodiploid (subG1) cells by flow cytometry. Electroporation of Kasumi-1 cells together with the RUNX1-CBFA2T1 siRNA siAGF1 neither increased the amount of hypodiploid cells nor the amount of annexin V positive cells when compared to control cells. Instead, after siAGF1 treatment we observed a slight but reproducible decrease in the amount of apoptotic cells in both assays. This decrease was more pronounced after two consecutive electroporations with siRNAs. Repeating the siAGF1 electroporation after 4 days resulted in a threefold decrease of the amount of annexin V stained cells (fig. 5) indicating that RUNX1-CBFA2T1 depletion has an antiapoptotic effect on Kasumi-1 cells, and that the reduced proliferation upon siRNA treatment is not related to increased apoptosis.

### RUNX1-CBFA2T1 siRNAs induce senescence in Kasumi-1 cells

**Figure 6:**
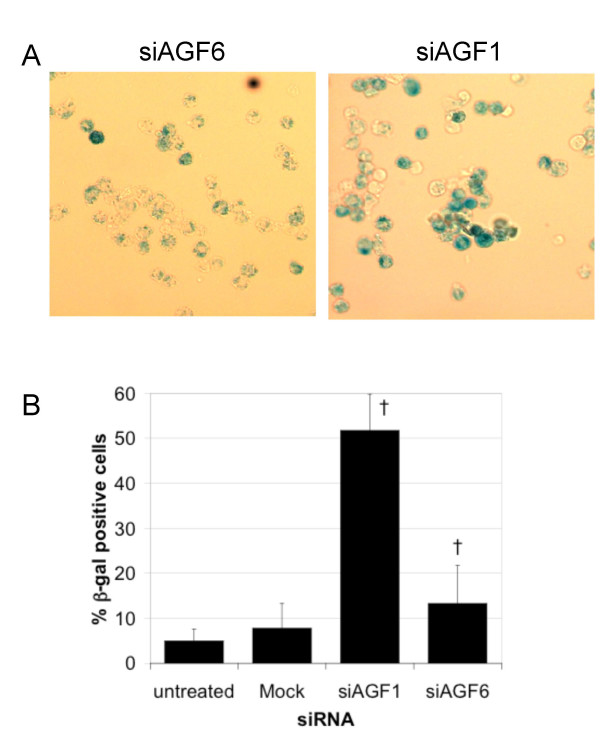
***RUNX1-CBFA2T1 siRNAs induce senescence in Kasumi-1 cells ***A. Senescence-associated β-galactosidase stained cells. Kasumi-1 cells were electroporated with siRNAs at days 0 and 4, and were examined at day 8 for senescence by staining for senescence-associated β-galactosidase. B. Quantification of cellular senescence. Cells were treated as above, and the ratio of β-galactosidase positive cells was examined by counting. Error bars represent the standard deviations of single-blinded counts performed by three different experimentators. †, p < 0.01 according to Student's t-test

The observed G1 arrest upon RUNX1-CBFA2T1 depletion could be either reversible or irreversible. An irreversible G1 arrest is a hallmark of cellular senescence. To address the question of the nature of the G1 arrest, and to analyze a possible influence of RUNX1-CBFA2T1 on cellular senescence, we stained siRNA- and mock-treated cells for senescence-associated β-galactosidase activity. After two or more electroporations with RUNX1-CBFA2T1 siRNA, but not with control siRNA, a significant fraction of the Kasumi-1 cells stain positive for β-galactosidase (fig. 6A). Cell counting revealed up to 50% of senescent cells, whereas mock or control siRNA treatment caused only a minor increase in senescent cells compared to untreated cells (fig. 6B). Therefore, depletion of RUNX1-CBFA2T1 results in an increase in cellular senescence. This implies that the observed G1 arrest is irreversible for a major fraction of RUNX1-CBFA2T1 siRNA treated cells.

### Haematopoetic growth factors cannot rescue RUNX1-CBFA2T1-depleted cells in the long term

**Figure 7:**
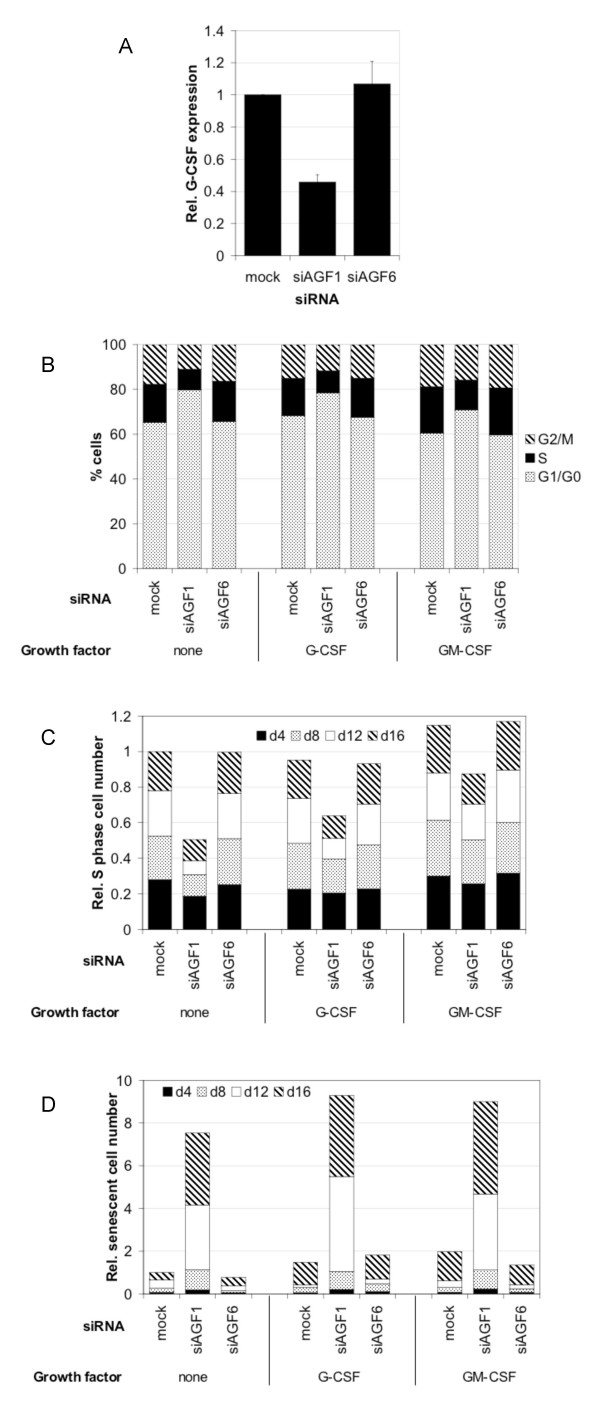
***Effects of growth factors on the cell cycle distribution and extent of senescence of siRNA-treated Kasumi-1 cells ***A. Autocrine G-CSF expression in dependence on the siRNA treatment. Kasumi-1 cells were electroporated at days 0 and 4. Total RNA was isolated on day 8 and analyzed by real-time RT-PCR. The columns and error bars represent the means and standard deviations of three independent experiments. B. Cell cycle distribution of siRNA-treated cells in the absence and presence of growth factors. Kasumi-1 cells were analyzed on day 8 by FACS analysis as described in Materials and Methods. C. Amount of S phase cells in dependence on the length of RUNX1-CBFA2T1 depletion. D. Amount of senescent cells dependence on the length of RUNX1-CBFA2T1 depletion. Growth factors are indicated below the graphs. The data shown in B, C and D were obtained from the same time course experiment. Cells were electroporated with the indicated siRNAs at days 0, 4, 8 and 12, and were analyzed on days 4, 8, 12 and 16.

A possible mechanism of how RUNX1-CBFA2T1 prevents cell cycle arrest and senescence is the autocrine production of growth factors. Kasumi-1 cells express, for instance, G-CSF (fig. 7A), but not GM-CSF (data not shown). Depletion of RUNX1-CBFA2T1 caused a twofold decrease in G-CSF transcript levels (fig. 7A). Therefore, we examined whether addition of haematopoetic growth factors rescued siRNA-treated cells from cell cycle arrest and senescence.

Depletion of RUNX1-CBFA2T1 for 16 days reduced the fraction of S phase cells twofold compared to control cells. This decrease is independent of G-CSF or GM-CSF (fig. 7B). When analyzing the cell cycle distribution siRNA or mock-treated cells in dependence on the duration of siRNA treatment, the fraction of S phase cells already decreases within 4 days of leukaemic fusion protein depletion and reaches its minimum after further 4 to 8 days (fig. 7C). Addition of G-CSF, and particularly of GM-CSF delayed this decrease, but could not prevent it. Furthermore, in RUNX1-CBFA2T1 siRNA treated cells, neither G-CSF nor GM-CSF addition caused any substantial change in the generation of senescent cells compared to cells without growth factors (fig. 7D). In each setting, the fraction of senescent cells increased from 2% at day 4 to 30–50% at days 12 and 16. Control groups contained between 1–10% of senescent cells.

**Figure 8:**
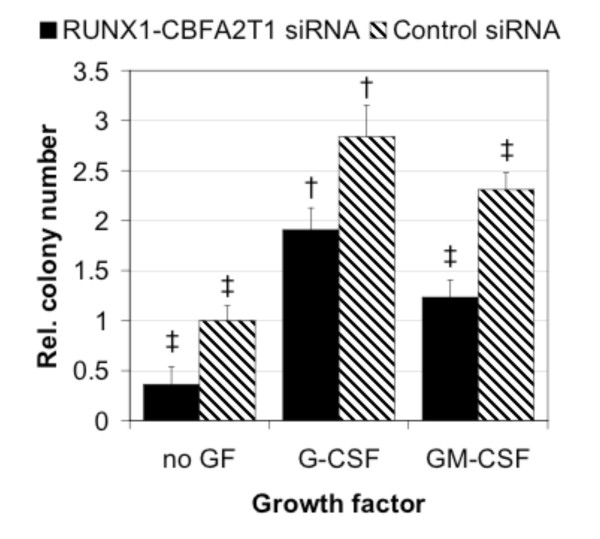
***Influence of haematopoetic growth factors on the colony formation of siRNA-treated Kasumi-1 cells ***Kasumi-1 cells were electroporated with the indicated siRNAs, plated in semi-solid medium containing the indicated growth factors, and analyzed as described in Material and Methods. The columns and error bars represent the mean values and standard deviations of four to five independent experiments. †, p < 0.01; ‡, p < 0.001 according to Student's T test.

Finally, we examined the effects of several haematopoetic growth factors on colony formation of siRNA-treated Kasumi-1 cells. Supplementing the semisolid medium with the corresponding growth factors increased colony numbers for both control cells and RUNX1-CBFA2T1-depleted cells (fig. 8). G-CSF enhanced colony formation by control cells twofold, and by RUNX1-CBFA2T1-depleted cells threefold, and GM-CSF three- and fivefold, respectively. However, in comparison to control cells, neither G-CSF nor GM-CSF could completely restore colony formation of RUNX1-CBFA2T1-depleted cells.

Taken together, the addition of G-CSF or GM-CSF delays, but does not prevent the G1 arrest in RUNX1-CBFA2T1-depleted cell. Furthermore, senescence is not inhibited by these growth factors. In line with these findings, neither of these growth factors completely restored the impaired clonogenicity of such cells. These results indicate that neither G-CSF nor GM-CSF can rescue leukaemic cells suffering of siRNA-mediated RUNX1-CBFA2T1 depletion.

## Discussion

The leukaemic fusion protein RUNX1-CBFA2T1 inhibits haematopoetic differentiation by repressing gene expression via recruitment of histone deacetylases [4-7], and by sequestering transcription factors such as C/EBPα, SMADs or vitamin D_3 _receptor [8-11]. In addition, RUNX1-CBFA2T1 inhibits cell proliferation by inducing apoptosis in a variety of different cell types [13,46], in spite of repressing the proapoptotic gene p14^Arf ^[17]. The notable exception are haematopoetic stem cells (HSCs); ectopic expression of RUNX1-CBFA2T1 causes an increased clonogenicity and results in their expansion both *ex vivo *and *in vivo *[20,21]. In addition to the block of differentiation, RUNX1-CBFA2T1 may also support proliferation, as, for instance, RUNX1-CBFA2T1-expressing HSCs maintain their telomere lengths and their CD34 expression [21].

We show that RUNX1-CBFA2T1 also supports proliferation, CD34 expression and colony formation in the t(8;21)-positive leukaemic cell line Kasumi-1. Thus, RUNX1-CBFA2T1 functions are similar in HSCs and in Kasumi-1. In the latter, support of proliferation is not caused by an inhibition of cell death. Instead, as observed with many other cell types [13,46], RUNX1-CBFA2T1 expression is associated with a certain extent of apoptosis. This observation disagrees with a report employing ribozymes to interfere with RUNX1-CBFA2T1 expression [47]. In contrast to our study, no clear reduction of either fusion transcript or protein was shown. It may, thus, be possible that the ribozyme effects observed in this study are not related to a suppression of RUNX1-CBFA2T1.

The reduced proliferation of Kasumi-1 cells upon RUNX1-CBFA2T1 depletion is paralleled by a cell cycle arrest in G1. Particularly from a therapeutic point of view, it is important to know, whether this G1 block will be relieved by recovering RUNX1-CBFA2T1 expression, or by growth factors, or whether this arrest is irreversible. We observe that RUNX1-CBFA2T1 depleted cells show enhanced levels of senescence-associated β-galactosidase activity, a hallmark of cellular senescence. Therefore, the inhibition of G1 to S transition is associated by the induction of senescence indicating that this G1 arrest is irreversible. Interestingly, the elevated levels of p27^Kip1 ^(CDKN1B) during RUNX1-CBFA2T1 depletion are in line with the recently demonstrated central role of this CDK inhibitor in the establishment of senescence [49,50].

Furthermore, RUNX1 was shown to induce senescence in murine embryonic fibroblasts, probably by inducing p19^Arf ^[17]. Since RUNX1-CBFA2T1 inhibits the expression of p19^Arf ^and of its human homologue, p14^Arf ^[17], an increased expression of this regulator of p53 may also account for the induction of senescence in RUNX1-CBFA2T1-depleted Kasumi-1 cells.

An important point to consider is a possible relation between the effects of RUNX1-CBFA2T1 on clonogenicity, proliferation and senescence, and its anti-differentiation activity. For instance, increased β-galactosidase activity may also be associated with late monocytic differentiation [48]. Moreover, later stages of myeloid differentiation are characterized by a stop of proliferation. Therefore, the observed inhibition of proliferation upon RUNX1-CBFA2T1 depletion might be directly related to myeloid differentiation. Kasumi-1 and SKNO-1 cells express the myeloid differentiation marker CD11b when treated with RUNX1-CBFA2T1 siRNA in combination with TGFβ and vitamin D_3 _[14]. However, in the absence of differentiation inducers, even extended siRNA treatment only results in a small fraction of less than 5% of CD11b-positive cells [14], and data not shown) which cannot be accountable for the up to 50% β-galactosidase positive cells. Nevertheless, depletion of RUNX1-CBFA2T1 causes an onset of myeloid differentiation; therefore, a possible link between the antiproliferative effects of RUNX1-CBFA2T1 suppression and early differentiation events cannot be entirely excluded.

The haematopoetic growth factors G-CSF and GM-CSF were shown to support clonogenicity and proliferation in Kasumi-1 cells [40]. However, neither of these factors prevented senescence in RUNX1-CBFA2T1-depleted cells. Furthermore, G1-S transition was only transiently restored; after extended terms of RUNX1-CBFA2T1 depletion, the fraction of S phase cells also diminished in the presence of G-CSF or GM-CSF. Finally, clonogenicity of RUNX1-CBFA2T1-depleted cells was not completely restored in the presence of G-CSF or GM-CSF. These findings show that at least some haematopoetic growth factors cannot rescue leukaemic cells treated with RUNX1-CBFA2T1 siRNAs, and suggest that targeting of leukaemic cells with such siRNAs *in vivo *might interfere with the development and maintenance of leukaemia. However, a remaining possibility might be that the stroma environment with its complex mixture of growth factors and cell-cell interactions may be able to rescue RUNX1-CBFA2T1-depleted cells. We are currently investigating this question both in cell culture and *in vivo*.

A major point of concern for siRNA applications is the specificity of the corresponding siRNA [51]. Reasons for "off-target" effects may be (i) the siRNA-induced RNA cleavage of transcripts with imperfect homology to the siRNA [52,53], (ii) a possible competition with endogenously expressed micro RNAs (miRNAs), which may be involved in the control of, for instance, cell differentiation, for RISC protein components [54], and (iii) the induction of an interferon response [45,55]. However, neither RUNX1-CBFA2T1 siRNAs nor the mismatch control siRNA affected the clonogenicity of five different leukaemic cell lines not carrying the translocation t(8;21). Furthermore, expression of the interferon-inducible gene STAT1 is not changed upon electroporation with RUNX1-CBFA2T1 siRNA or mismatch control siRNA. These results do not argue for general "off-target" effects being responsible for the observed changes in cell proliferation and clonogenicity of RUNX1-CBFA2T1 siRNA-treated Kasumi-1 cells. Instead, the data suggest the siRNA-mediated depletion of this leukaemic fusion protein as the cause for the observed phenotypic changes.

## Conclusions

In summary, we show that RUNX1-CBFA2T1 does not only inhibit the myeloid differentiation [14], but is also essential for the proliferation and clonogenicity of the t(8;21)-positive leukaemic cell line Kasumi-1 by interfering with the establishment of senescence. Since this cell line was derived from a patient suffering of leukaemia refractory to chemotherapy [40], these findings suggest a central role of RUNX1-CBFA2T1 not only in the expansion of preleukaemic progenitor cells, but also in the maintenance of the leukaemia. Moreover, they imply that this leukaemic fusion gene is a promising target for molecularly defined therapeutic approaches.

## Competing interests

HPV is an employee of Alnylam Europe AG, which also provided some of the siRNAs used in this study.

## Authors' contributions

NM performed the proliferation, senescence and cell cycle assays. BD analyzed the clonogenicity of growth-factor treated Kasumi-1 cells and of t(8;21) negative cell lines. HR performed the immunoblot analyses and was involved in the analysis of clonogenicity and apoptosis. CC examined the expression levels of CD34 and of STAT1. HPV, AG, GH and AN participated in the coordination of the study. JK and OH conceived and designed the experiments. All authors read and approved the final manuscript.

## Pre-publication history

The pre-publication history for this paper can be accessed here:
